# Comprehensive analysis of the role of diverse programmed cell death patterns in sepsis

**DOI:** 10.3389/fimmu.2025.1685533

**Published:** 2025-11-19

**Authors:** Xiaotongning Yu, Zihao Zhou, Jing Ma, Xiaonan Wang, Chunyu Zhao, Anqi Hou, Shuo Sun

**Affiliations:** 1 School of Life Sciences, Jining Medical University, Rizhao, China; 2 Zhejiang Hangzhou High School, Hangzhou, China; 3 Department of Cardiology, Sir Run Run Shaw Hospital, School of Medicine, Zhejiang University, Hangzhou, China

**Keywords:** sepsis, programmed cell death, transcriptome profiling, ScRNA-seq, cell communication

## Abstract

**Background:**

Sepsis, a life-threatening condition with persistently high mortality, involves dysregulated immune responses and programmed cell death (PCD). However, the specific roles and interactions of diverse PCD pathways in sepsis pathogenesis remain incompletely understood. This study aimed to systematically characterize PCD patterns and their clinical relevance in sepsis.

**Methods:**

We integrated three bulk transcriptomic datasets (81 controls, 165 sepsis patients) and one single-cell RNA sequencing (scRNA-seq) dataset (4 controls, 4 early sepsis patients, 52,315 cells) from public databases. Gene set variation analysis (GSVA) quantified activity of 13 PCD pathways. Immune infiltration was assessed via single-sample gene set enrichment analysis (ssGSEA). A cell death-associated signature (CDS) risk score was developed using least absolute shrinkage and selection operator (LASSO) regression. scRNA-seq analysis identified cell-type-specific PCD activation and intercellular communication using Seurat, AUCell, and CellPhoneDB. Additionally, an independent RNA-seq cohort generated from our own sequencing of sepsis patients and healthy controls was used for external validation.

**Results:**

Transcriptomic analysis identified 5,591 differentially expressed genes enriched in immune and cell death pathways. Four PCD pathways-ferroptosis, disulfidptosis, NETosis, and entotic cell death-were significantly upregulated in sepsis and strongly correlated with immune cell infiltration, such as activated dendritic cells and neutrophils. The CDS risk score, based on 18 core PCD genes, showed excellent diagnostic accuracy across both public microarray datasets (AUC = 0.961 and 0.844) and our independent high-throughput RNA-seq dataset (AUC = 0.975). scRNA-seq revealed monocytes as dominant effectors, exhibiting heightened activation of ferroptosis, entotic death, and netotic pathways alongside metabolic reprogramming, including enhanced glutathione metabolism and oxidative phosphorylation (OXPHOS). Furthermore, monocyte-centric intercellular communication was dysregulated in sepsis, featuring upregulated MIF-CXCR4, ANXA1-FPR2, and HLA-KIR signaling axes.

**Conclusions:**

By integrating public microarray and single-cell transcriptomic data with independent high-throughput sequencing validation, this study analysis identifies ferroptosis, disulfidptosis, netotic death, and entotic death as key dysregulated PCD pathways in sepsis, with monocytes serving as central hubs integrating PCD, metabolic reprogramming, and immune communication. The CDS risk score provides a robust diagnostic and stratification tool. Targeting monocyte-driven PCD-metabolism-communication networks offers promising avenues for precision immunotherapy in sepsis.

## 1 Introduction

Sepsis is a life-threatening organ dysfunction caused by a dysregulated host response to infection, with persistently high morbidity and mortality rates, posing a major global public health challenge (1). Epidemiological studies suggest an increasing global incidence of sepsis, with mortality rates exceeding 50% once the condition escalates to septic shock (2). The pathophysiology of sepsis is complex and multifactorial, involving dysregulated innate and adaptive immune responses, aberrant inflammatory signalling, metabolic reprogramming, and endothelial dysfunction (3–6). Despite recent advancements in therapeutic approaches, considerable obstacles persist in the realms of early diagnosis, disease evaluation, and targeted treatment of sepsis.

Programmed cell death (PCD) encompasses a spectrum of genetically controlled mechanisms that maintain tissue homeostasis, regulate immune responses, and eliminate damaged or infected cells (7). Since the introduction of the concept of apoptosis in 1972 (8), a growing number of distinct modalities of PCD have been identified, including ferroptosis, pyroptosis, and necroptosis, among others; to date, at least 13 PCD pathways have been recognized (9). In the context of sepsis, dysregulated PCD has been associated with immune cell dysfunction, organ damage, and the progression of the disease. For example, excessive apoptosis during sepsis can result in the depletion of immune cells, thereby compromising the host’s immune defense, while necroptosis and similar forms of cell death may intensify inflammatory responses and contribute to additional organ injury (10, 11).

In recent years, the rapid advancement of high-throughput sequencing technologies has established both bulk and single-cell RNA sequencing (scRNA-seq) as powerful methodologies for exploring the pathological mechanisms underlying sepsis (12). Transcriptomic profiling enables a comprehensive analysis of gene expression alterations, which aids in the identification of critical genes and signaling pathways associated with sepsis. Concurrently, scRNA-seq offers insights at the single-cell level, revealing cellular heterogeneity and allowing for a detailed examination of the roles and molecular mechanisms of specific cell types in the context of sepsis (13). Previous investigations utilizing these technologies on peripheral blood mononuclear cells (PBMCs) from sepsis patients have uncovered significant changes in cellular composition, gene expression, and signaling pathways, providing valuable insights into disease mechanisms and informing clinical interventions (14). Nevertheless, a thorough understanding of the interplay between sepsis and PCD-related pathways, as well as the roles of these pathways across various immune cell types, remains insufficiently explored.

This study aims to systematically investigate the associations between sepsis and 13 known PCD pathways by integrating transcriptomic and scRNA-seq. We seek to identify key cell death–related genes and critical immune cell populations, elucidate their functional roles in the pathogenesis of sepsis, and construct a robust cell death–associated signature (CDS) risk score. These efforts aim to provide novel insights and potential biomarkers for early diagnosis, disease stratification, and precision therapy in sepsis.

## 2 Methods

### 2.1 Public transcritome sequencing data and PCD-related genes collection

We conducted the primary analysis using public microarray-based mRNA expression profiles obtained from the GEO database (https://www.ncbi.nlm.nih.gov/geo/) (GSE57065, GSE54514, and GSE28750), comprising a total of 81 samples in the control group and 165 samples in the sepsis group, all of which were derived from human blood. More details of the collected datasets are presented in **Supplementary Table 1**. To investigate PCD, we integrated key regulatory genes collected from the KEGG database (15), GeneCards (16), MSigDB (17), Reactome (18), and relevant review articles. This includes genes associated with various cell death pathways: alkaliptosis (8 genes), apoptosis (655 genes), autophagy (461 genes), cuproptosis (14 genes), disulfidptosis (4 genes), entotic cell death (16 genes), ferroptosis (97 genes), lysosome-dependent cell death (447 genes), necroptosis (179 genes), NETosis (16 genes), oxeiptosis (23 genes), parthanatos (9 genes), and pyroptosis (52 genes). In total, 1,981 PCD-related genes were compiled. The final gene lists for each PCD subtype are provided in **Supplementary Table 2**.

### 2.2 Microarray data preprocessing

The gene expression was detected by Affymetrix Genome U133 Plus 2.0 Array (GSE57065 and GSE28750) and Illumina HumanHT-12 V3.0 expression beadchip (GSE54514). Gene probes were annotated as gene symbols. Probes without matching gene symbols and matching multiple symbols were excluded. Gene expression value of duplicate gene symbol was calculated as the median value. Batch effects among the three training datasets were corrected using the ComBat function from the “sva” R package with default parameters, specifying the batch variable as the dataset ID (19). Then the effectiveness of batch correction was assessed with principal component analysis (PCA) plots.

### 2.3 Collection of blood samples from sepsis patients and healthy individuals

To obtain human peripheral blood monocytes for further RNA sequencing, a total of 18 participants were enrolled in this study, including 10 sepsis patients and 8 non-sepsis controls. Eligible participants met the following criteria: aged between 18 and 85 years. For sepsis cases, fulfillment of the Sepsis-3 definition of sepsis. For non-sepsis controls, volunteers were recruited without a diagnosis of sepsis (**Supplementary Table 3**).

### 2.4 RNA-Seq library preparation and sequencing

RNA purification, reverse transcription, library construction and sequencing were conducted at Shanghai Majorbio Biopharm Technology Co., Ltd.(Shanghai, China). Total RNA from PBMCs was extracted using TRIzol reagent and quantified with a NanoDrop spectrophotometer. Libraries were prepared using 1ug of total RNA and sequenced on the NovaSeqX Plus platform (PE150). Briefly, messenger RNA was isolated using polyA selection with methodbyoligo (dT) beads and then fragmented. Double-stranded cDNA was synthesized using a SuperScript double-stranded cDNA synthesis kit (Invitrogen, CA) using random hexamer primers. The synthesized cDNA was subjected to end-repair, phosphorylation, and adapter ligation, according to the library construction protocol. Libraries were size selected for300 bp cDNA fragments using 2% Low Range Ultra Agarose, followed by PCR amplified with Phusion DNA polymerase (NEB) for 15 cycles. The sequencing library was quantified with Qubit 4.0 and sequenced using the NovaSeq Reagent Kit on NovaSeq X Plus platform (PE150).

### 2.5 Analysis of bulk RNA-seq data

Initial reads QC metrics (base quality distribution) were assessed using FASTQC (http://www.bioinformatics.babraham.ac.uk/projects/fastqc/). NGS QC (20) toolkits were used to trim adaptors and low-quality reads. The clean reads were mapped to the human (hg19) genomes using HISAT2 version 2.2.1 (21) with default settings. BAM files containing uniquely mapped reads were used as inputs for the Stringtie (22), and transcripts per million reads values were calculated to quantify gene expression levels. Differential gene expression analysis was performed using the DESeq2 package (23) in R.

### 2.6 Pathway and functional enrichment analysis

We utilized the R package “clusterProfiler” (24) to conduct kyoto encyclopedia of genes and genomes (KEGG) (25) and gene ontology (GO) (26) enrichment analyses. Additionally, we performed gene set enrichment analysis (GSEA) (27) to identify the underlying pathways, with the threshold for significant terms being adjusted p-value <0.05.

### 2.7 Pathway activity calculation

To investigate potential pathway-level changes in gene expression from microarray-based mRNA expression profiles, we employed a non-parametric and unsupervised gene set variation analysis (GSVA) to assess pathway enrichment results from the sequencing data. GSVA enables the evaluation of pathway activity variations across individual samples. We conducted the analysis using the GSVA package in R software and calculated the enrichment scores of pathways in all samples to identify any differences in pathway activity across sample (28). For pathway activity analysis of cell clusters derived from scRNA-seq data, we first aggregated single-cell expression profiles into pseudobulk expression matrices by averaging gene expression within each annotated cell type. Genes with zero expression across all cells were excluded. Using these pseudobulk matrices, we applied GSVA to calculate enrichment scores for 13 programmed cell death (PCD) pathways across cell types. In parallel, we utilized the “AUCell” package to compute cell-level activation scores for key PCD pathways. AUCell uses a rank-based scoring method to calculate the activity level of gene sets and computes a gene set activation score for each cell by utilizing the area under the curve (AUC) (29).

### 2.8 Development and validation of the CDS risk score

The least absolute shrinkage and selection operator (LASSO) (30) was used to construct the optimal CDS risk score in sepsis. To ensure the independence of training and validation, three GEO datasets (GSE57065, GSE54514, GSE28750) were combined as the training set (n=246; 81 healthy controls and 165 sepsis patients), and GSE69528 (n=124; 22 healthy controls and 102 sepsis patients) was used as an independent validation set.

Candidate genes were first obtained by intersecting PCD-related genes with differentially expressed genes between sepsis and control groups. A random forest algorithm was then applied on the training set only to narrow down the feature set, and genes with high importance scores were retained. Subsequently, LASSO regression with 10-fold cross-validation and a binomial family (logistic regression), alpha=1, and set the random seed (3699) to ensure reproducibility was performed in the training set to identify the optimal gene set and estimate the coefficients for the risk score model. The λ value that minimized partial likelihood deviance (λ_min) was used to fit the final model.

We created a receiver operating characteristic (ROC) curve to evaluate the predictive capacity of 13 PCD pathways, core death genes, and CDS risk score in both the training and validation cohorts. The AUC value was calculated using the “pROC” package (31). The “rms” and “dcurves” R packages were used to conduct calibration curve analysis and decision curve analysis.

### 2.9 Immune infiltration analysis

We utilized single-sample gene set enrichment analysis (ssGSEA), a novel method for gene enrichment, to compare the immune score of each sample. Feature gene panels for each immune cell type were acquired from the published literature (32, 33). The GSVA R package (version 1.48.2) was used to convert the gene expression matrix into an enrichment score matrix based on immune cell–specific gene sets. Subsequently, differences in immune cell activity between groups (sepsis vs. control) were compared using the Wilcoxon rank-sum test. Spearman correlation coefficients were calculated to assess the relationships between different immune cell types, and appropriate adjustments were made for multiple comparisons in the statistical analyses. All statistical analyses were performed using R software (version 4.4.1, https://www.r-project.org), and all p-values were two-sided. A p-value < 0.05 was considered statistically significant.

### 2.10 scRNA-seq analysis

The scRNA-seq dataset used in this study included four control and four early sepsis samples, generating a total of 52,315 cells (GSE217906). Cells were retained with expressed > 300 genes, mitochondrial gene expression < 21%, hemoglobin gene expression < 5%, and nCount_RNA > 200. In addition, cells with nCount_RNA in the top 3% were excluded to avoid potential doublets or outliers. Next, the main analysis was implemented through Seurat v5.2.1 (13). The gene expression matrix was normalized via the “NormalizeData” function, followed by the identification of highly variable genes using “FindVariableFeatures”. Batch effects were addressed through canonical correlation analysis. Dimensionality reduction was conducted using PCA, and the top 20 principal components, selected based on the “ElbowPlot” function, were retained for downstream analysis. Cell clustering was carried out with the “FindNeighbors” and “FindClusters” functions (resolution = 1), and visualized using uniform manifold approximation and projection (UMAP). Cluster-specific DEGs were identified via the “FindAllMarkers” function and annotated based on known canonical markers. Cells expressing markers of more than one cell type were defined as doublets and excluded from further analyses. To assess metabolic pathway activity at the single-cell level, we applied the “scMetabolism” package (34), which leverages the preloaded KEGG database and the Vision algorithm for quantification. The results were displayed using dot plots. CellChat was used to investigate molecular-level intercellular communication with default parameters (35).

## 3 Results

### 3.1 Transcriptomic characterization of PCD pathways in sepsis

To investigate the transcriptomic features of PCD pathways in sepsis, we integrated three publicly available transcriptomic datasets of sepsis and healthy controls. Batch effects were removed using the SVA algorithm (36), followed by dataset merging and normalization. PCA and box plots (**Supplementary Figure 1**) confirmed effective batch correction and data consistency.

**Figure 1:**
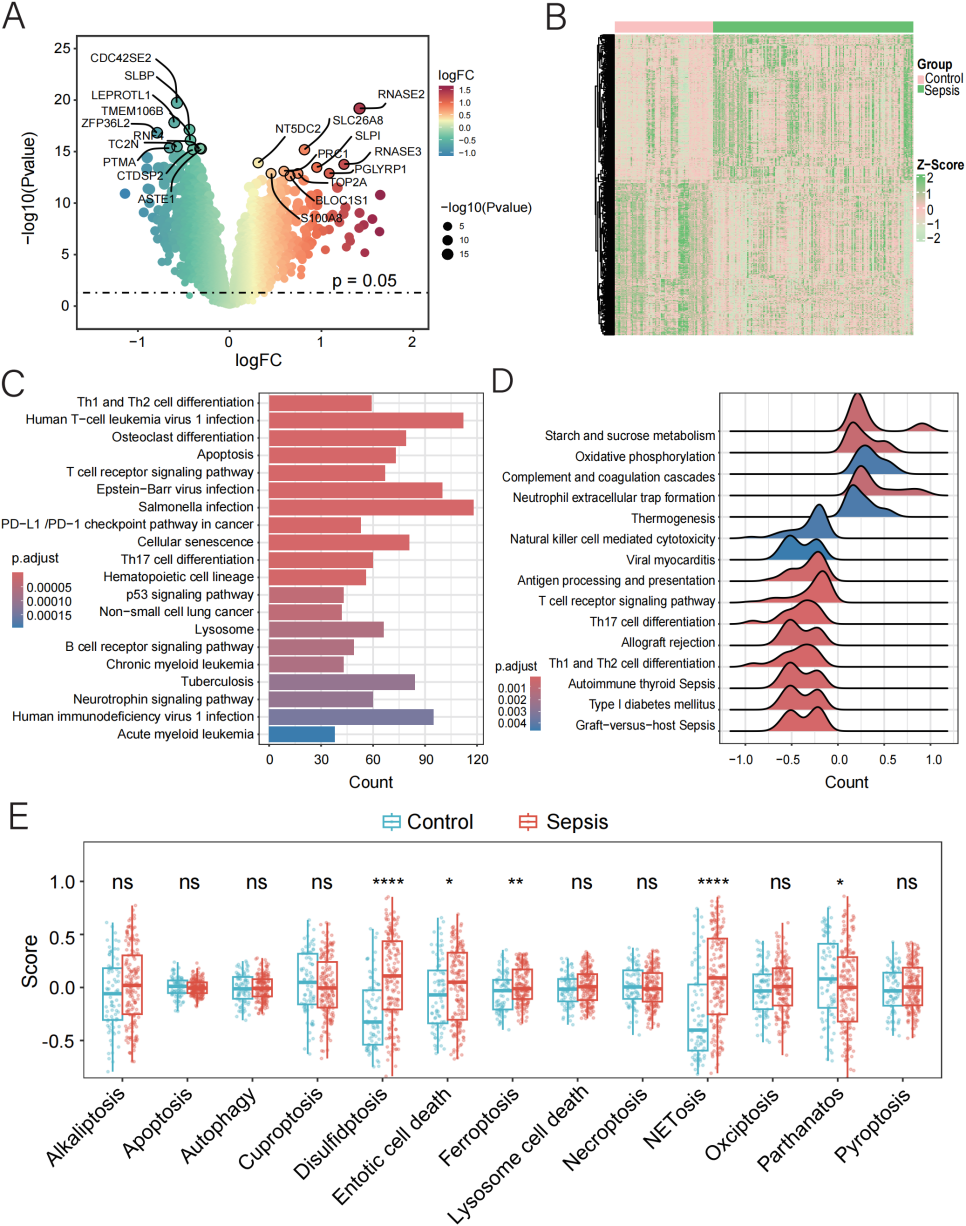
Function and pathway enrichment analysis of DEGs in control and sepsis groups. **(A, B)** Volcano and the heatmap plot of the DEGs from control and sepsis groups. **(C)** KEGG enrichment analyses based on the DEGs between control and sepsis groups. **(D)** GSEA analysis of KEGG pathways. **(E)** GSVA analysis of 13 PCD pathways demonstrated by box plots in control and sepsis groups.

Based on the preprocessed data, we identified 5,591 DEGs between sepsis and normal samples (p < 0.05), as illustrated by the volcano plot and heatmap (**Figures 1A, B**, **Supplementary Table 4**). Pathway enrichment analysis showed that these DEGs were enriched in key pathways involved in sepsis, including “Th1 and Th2 cell differentiation”, “T cell receptor signaling pathway”, and “PD-L1 checkpoint pathway in cancer”, as well as cell death-related pathways such as “Apoptosis”, “Cellular Senescence”, and the “p53 Signaling Pathway”. Infection-related pathways like “Human T-cell Leukemia Virus 1 Infection”, “Epstein–Barr Virus Infection”, and “Tuberculosis” were also enriched (**Figure 1C**). GSEA further revealed activation of immune-related pathways, including “Th17 Cell Differentiation”, “Antigen Processing”, “NET Formation” and suppression of metabolic and effector pathways, such as “Oxidative Phosphorylation” and “NK Cell Cytotoxicity” (**Figure 1D**). These findings suggest that sepsis may involve immune dysregulation, metabolic disturbance, and abnormal cell death.

To further characterize the involvement of PCD in sepsis, we applied GSVA to quantify the activity of 13 PCD-related pathways. Among these, four pathways—disulfidptosis, entotic cell death, ferroptosis, and NETosis—were upregulated in sepsis, while parthanatos (PARP-1-dependent cell death) showed reduced activity (**Figure 1E**, **Supplementary Table 5**). These results highlight the potential contribution of dysregulated PCD to the pathogenesis and progression of sepsis.

### 3.2 Linking immune infiltration and PCD in sepsis for diagnostic insight

**Figure 2:**
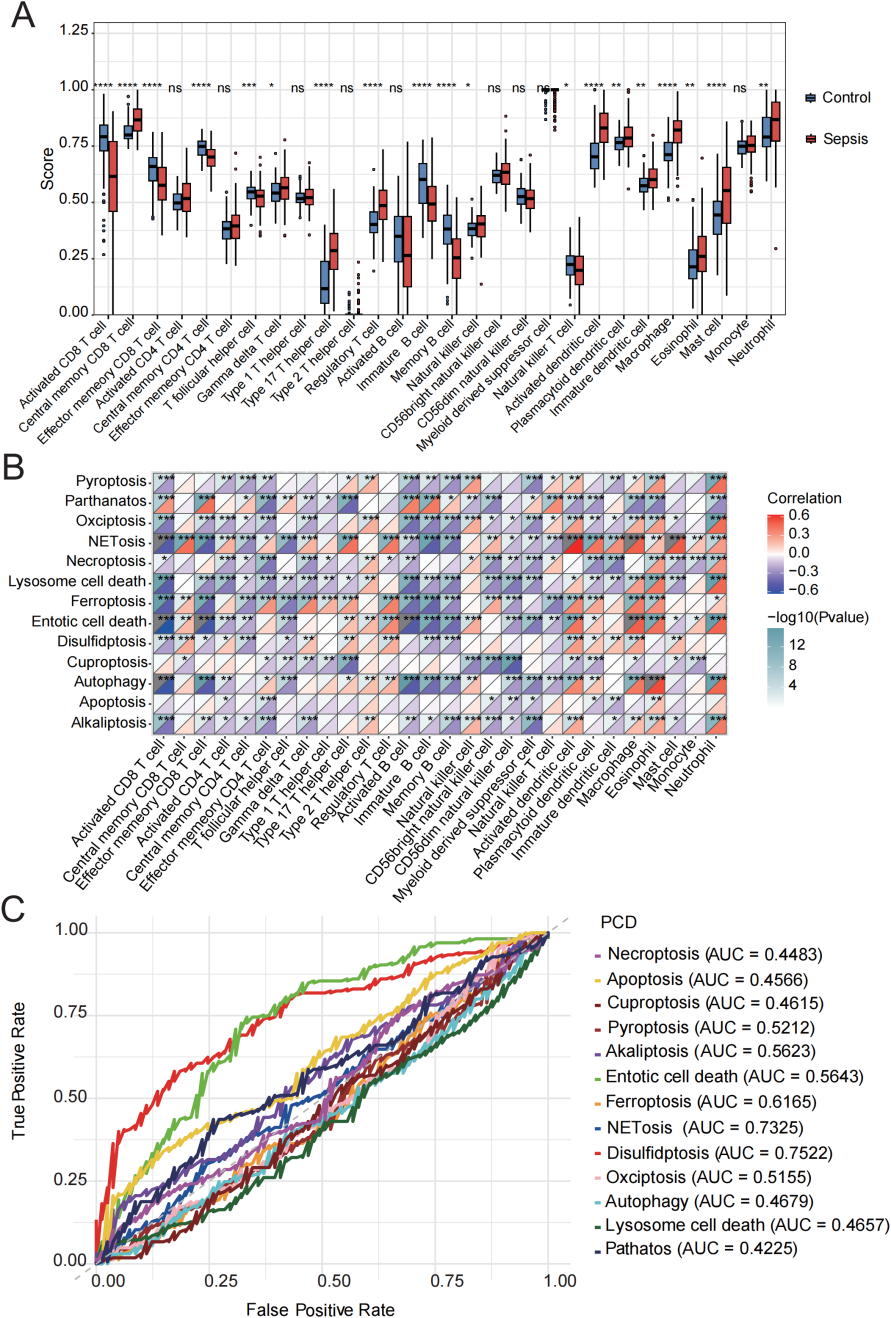
Immune cell infiltration evaluation. **(A)** Immune cell scores were assessed using ssGSEA in the control and sepsis groups. **(B)** Correlation analysis was performed between the GSVA scores of 13 PCD-related pathways and different immune cells. **(C)** ROC curve of the 13 PCD pathways in sepsis. ns, not significant, *p < 0.05, **p < 0.01, ***p < 0.001, ****p < 0.0001.

Stimulus-induced cell death may occur in a manner that alerts the immune system, thereby initiating immune responses against antigens derived from dead cells (37). The initiation and progression of sepsis are closely associated with extensive immune cell infiltration. Therefore, using the ssGSEA algorithm, we compared the immune infiltration profiles between normal and septic groups. Immune infiltration analysis revealed significant alterations in 19 out of 28 immune cell types. Most of the dysregulated immune cells, such as central memory CD8 T cells, natural killer cells, activated dendritic cells, and mast cells, were upregulated in sepsis (**Figure 2A**, **Supplementary Table 6**), indicating substantial changes in the immune microenvironment during the progression of the disease.

Subsequently, we conducted a correlation analysis between GSVA scores of 13 PCD pathways and the infiltration levels of 28 immune cell populations. The activities of parthanatos, NETosis, ferroptosis, entotic cell death, and disulfidptosis were found to be significantly positively correlated with the infiltration of several immune cell types, including central memory CD8 T cells, regulatory T cells, activated dendritic cells, macrophages, eosinophils, mast cells, and neutrophils. In contrast, alkaliptosis, lysosome-dependent cell death, and cuproptosis were significantly negatively associated with the majority of immune cell types (**Figure 2B**, **Supplementary Table 7**).

In addition, we evaluated the diagnostic performance of the 13 PCD-related GSVA scores in septic patients using ROC curve analysis (**Figure 2C**). Among these, the top five PCD pathways ranked by AUC were disulfidptosis (AUC = 0.75), NETosis (AUC = 0.73), ferroptosis (AUC = 0.62), entotic cell death (AUC = 0.56), and alkaliptosis (AUC = 0.56). Based on these findings, we identified the activation of NETosis, ferroptosis, entotic cell death, and disulfidptosis in the sepsis group, all of which were strongly associated with immune cell infiltration. Moreover, these PCD pathways demonstrated relatively high diagnostic efficacy, suggesting that they represent key mechanisms warranting further investigation in future studies.

### 3.3 Development of a cell death-associated risk score model for sepsis

**Figure 3:**
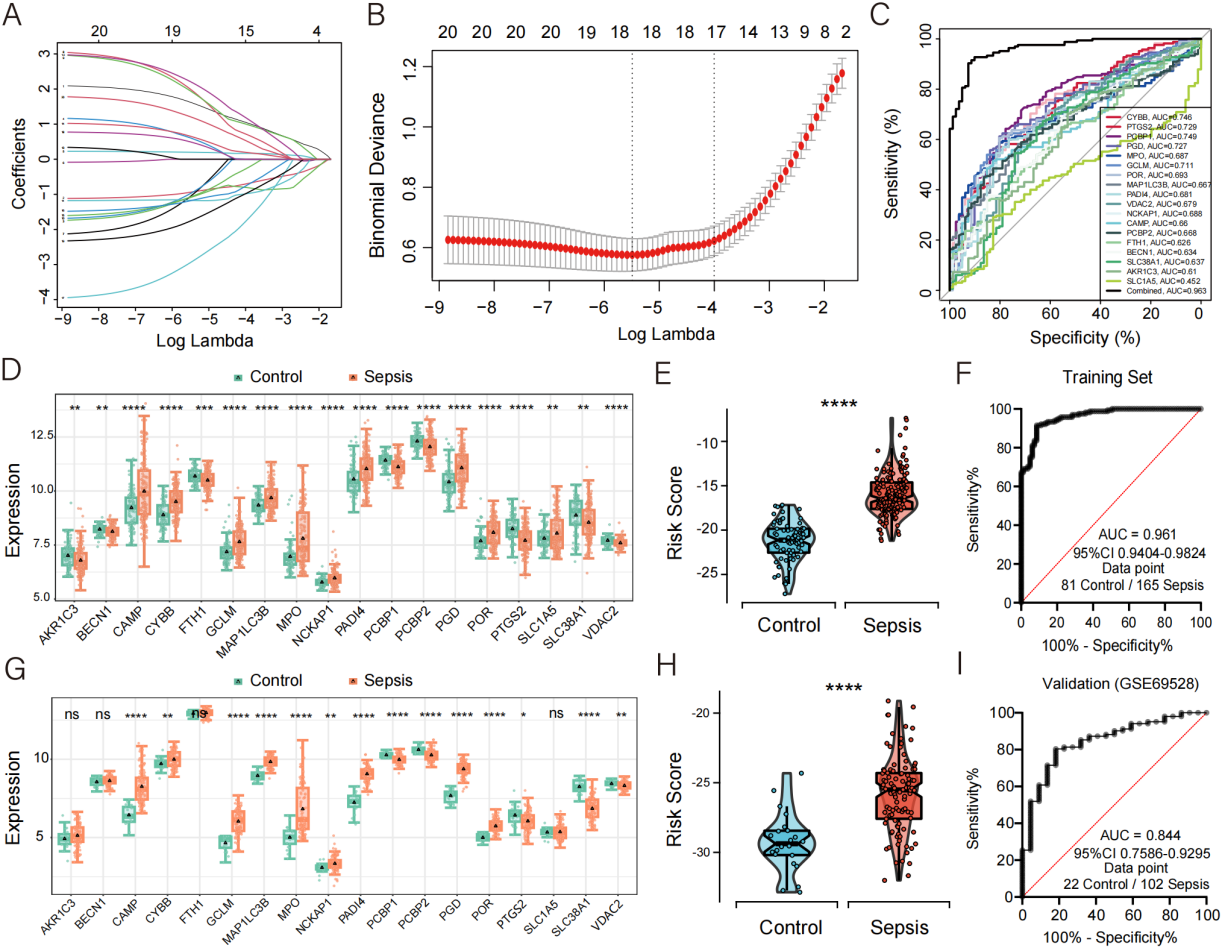
Development of CDS risk score based on core death genes. **(A)** Distribution of LASSO coefficients for feature genes. **(B)** Ten-fold cross-validation for parameter selection in the LASSO model. **(C)** ROC curve analysis of the 18 core death genes in sepsis. **(D, G)** Box plots showing the expression of death genes in control and sepsis patients, with **(G)** representing data from an external validation cohort. **(E, H)** Violin plot showing the distribution of CDS risk scores between control and sepsis patients, with **(H)** representing data from an external validation cohort. **(F, I)** ROC curve analysis of the CDS risk score in sepsis, with **(I)** representing data from an external validation cohort. ns, not significant, *p < 0.05, **p < 0.01, ***p < 0.001, ****p < 0.0001.

By intersecting genes involved in four PCD pathways—NETosis, ferroptosis, entotic cell death, and disulfidptosis—with the DEGs between septic patients and healthy controls in the training set (GSE57065, GSE54514, GSE28750), a total of 120 candidate genes were identified. Using a random forest algorithm, 20 genes were selected (Gini > 2.0) (**Supplementary Figure 2**). Subsequently, a LASSO regression analysis (**Figures 3A, B**) was performed on the training set, and 18 core death-related genes (AKR1C3, BECN1, CAMP, CYBB, FTH1, GCLM, MAP1LC3B, MPO, NCKAP1, PADI4, PCBP1, PCBP2, PGD, POR, PTGS2, SLC1A5, SLC38A1, VDAC2) were identified as having the minimum partial likelihood deviance. Based on these genes, the following CDS risk score formula was established:

CYBB * (1.6196034) + PTGS2 * (-0.9390386) + PCBP1 * (-1.0863174) + PGD * (0.6020607) + MPO * (0.2001920) + GCLM * (1.0476006) + POR * (1.9875715) + MAP1LC3B * (1.9357273) + PADI4 * (-0.9704860) + VDAC2 * (-1.1710069) + NCKAP1 * (2.2052000) + CAMP * (0.6512673) + PCBP2 * (-1.0707485) + FTH1 * (-1.1794027) + BECN1 * (-2.9384124) + SLC38A1 * (0.4903299) + AKR1C3 * (-1.7556165) + SLC1A5 * (1.2059844).

To assess the diagnostic potential of these 18 genes in sepsis, ROC curve analysis was performed, revealing high diagnostic accuracy (**Figure 3C**). Compared with healthy controls, the expression levels of CAMP, CYBB, GCLM, MAP1LC3B, MPO, NCKAP1, PADI4, PGD, POR, and SLC1A5 were significantly upregulated in septic patients (**Figure 3D**).

The diagnostic performance of this PCD-based signature was further validated in septic patients and demonstrated excellent diagnostic accuracy, with a precision of 0.961 (**Figures 3E, F**). To further confirm the robustness of the model, we validated its performance using the external dataset GSE69528. The expression levels of CAMP, CYBB, GCLM, MAP1LC3B, MPO, NCKAP1, PADI4, PGD, and POR were significantly upregulated in sepsis patients compared to controls (**Figure 3G**), which was consistent with the findings in the discovery cohort. Moreover, the validation cohort achieved an AUC of 0.844, highlighting the strong diagnostic performance of the model (**Figures 3H–I**). To further evaluate the calibration performance and clinical applicability of the model, we conducted calibration curve analysis (CCA) and decision curve analysis (DCA) on the training set and validation set, respectively. The results showed that both sets exhibited good performance (**Supplementary Figure 3**). In addition, we used another dataset GSE185263, which includes clinical information such as sequential organ failure assessment (SOFA) score and 28 day survival status, to plot ROC curves to evaluate the predictive effect of risk scores on SOFA and 28 day mortality outcomes. The results indicate that the risk score has a decent predictive ability for SOFA (AUC = 0.706), but its predictive effect on 28 day mortality is poor (AUC = 0.586) (**Supplementary Figure 4**).

### 3.4 Independent validation of the CDS model using an in-house high-throughput RNA-seq cohort

**Figure 4:**
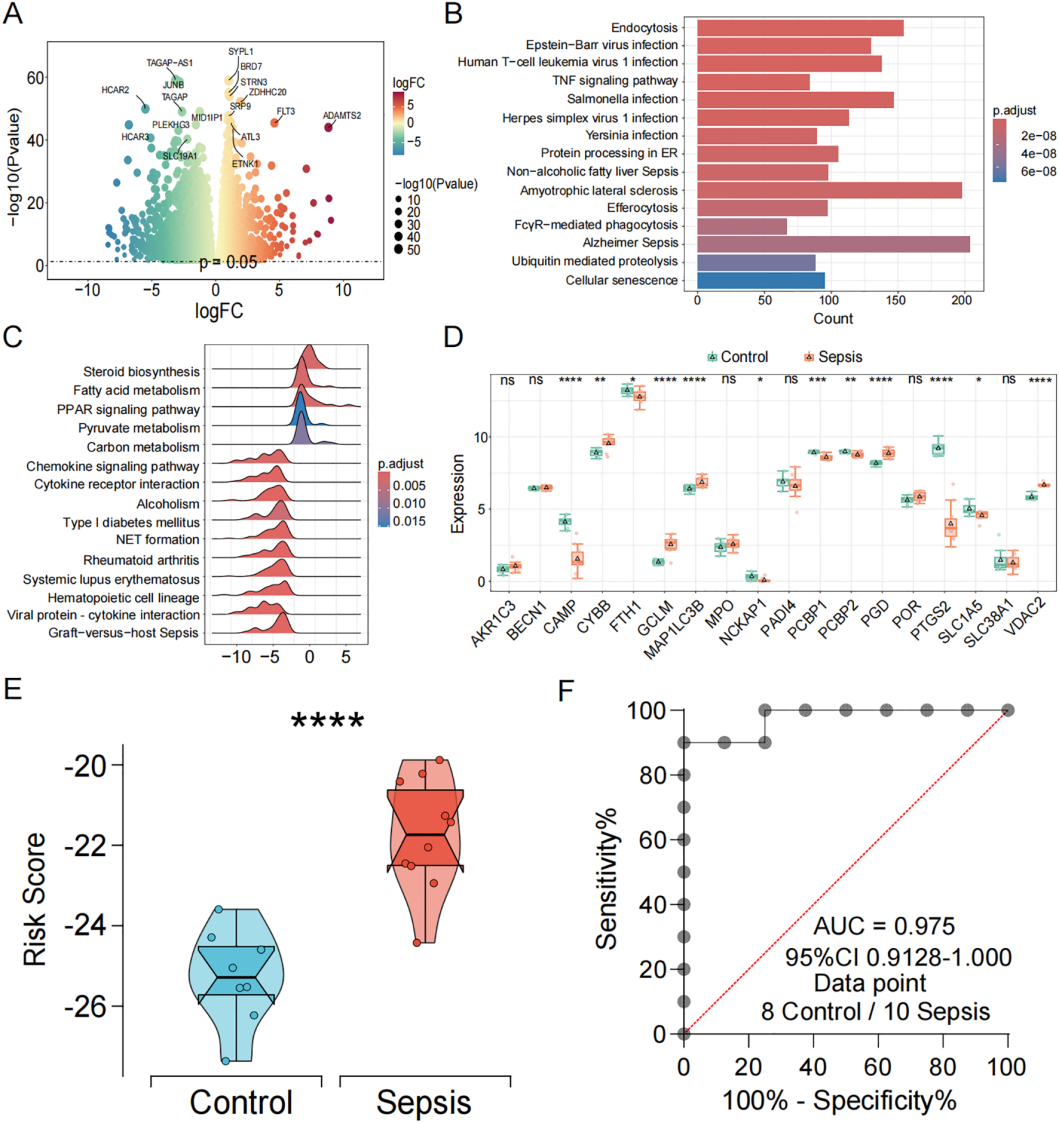
Independent transcriptomic validation and diagnostic evaluation of the CDS model in sepsis. **(A)** Volcano and the heatmap plot of the DEGs from control and sepsis groups. **(B)** KEGG enrichment analyses based on the DEGs between control and sepsis groups. **(C)** GSEA analysis of KEGG pathways. **(D)** Box plots showing the expression of death genes in control and sepsis patients. **(E)** Violin plot showing the distribution of CDS risk scores between control and sepsis patients. **(F)** ROC curve analysis of the CDS risk score in sepsis. ns, not significant, *p < 0.05, **p < 0.01, ***p < 0.001, ****p < 0.0001.

To independently validate the transcriptomic features and diagnostic efficacy of the CDS model, we generated and analyzed an in-house high-throughput RNA-seq dataset. Transcriptomic profiling of this cohort identified a total of 11,246 differentially expressed genes (DEGs) (p < 0.05) (**Figure 4A**, **Supplementary Table 8**). KEGG pathway enrichment analysis revealed that these DEGs were significantly enriched in “immune- and inflammation-related pathways”, “endocytosis”, “human T-cell leukemia virus 1 infection”, and “TNF signaling pathway” (**Figure 4B**). Gene set enrichment analysis (GSEA) further demonstrated significant enrichment of metabolic and immune-inflammatory pathways, such as the “PPAR signaling pathway”, “fatty acid metabolism”, “neutrophil extracellular trap formation”, and “cytokine–cytokine receptor interaction” (**Figure 4C**). Notably, the expression patterns of core PCD-related genes in this cohort were highly consistent with those observed in the discovery datasets, with genes such as CYBB, GCLM, MAP1LC3B, NCKAP1, and PGD markedly upregulated in sepsis patients (**Figure 4D**). Furthermore, the CDS risk score achieved an AUC of 0.975 in this independent cohort (**Figures 4E, F**), underscoring the model’s outstanding performance in high-throughput sequencing data and the robustness of its diagnostic capability.

### 3.5 Discovery of DEGs stratified by CDS risk score

**Figure 5:**
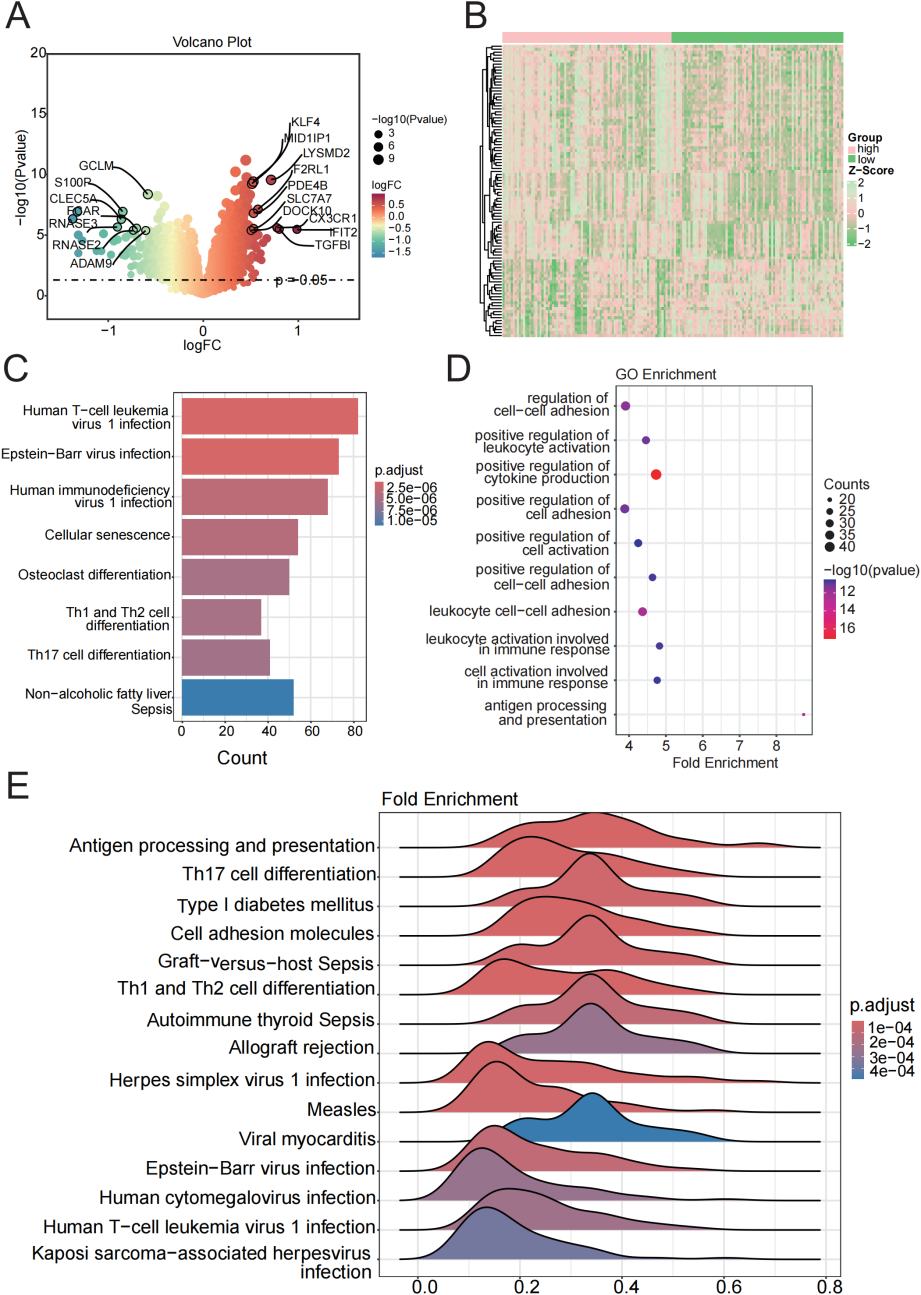
Enrichment analysis of functions and pathways in DEGs from low-risk and high-risk groups. **(A, B)** Volcano and heatmap plot of the DEGs between the low-risk and high-risk groups. **(C)** KEGG pathway between the low-risk and high-risk groups. **(D)** GSEA analysis of KEGG pathways. **(E)** GO terms enriched in the DEGs between the low-risk and high-risk groups.

Based on the median value of the CDS risk score, sepsis patients were stratified into high-risk and low-risk groups. A total of 3,350 DEGs (p < 0.05) were identified between the two groups (**Figures 5A, B**, **Supplementary Table 9**). KEGG enrichment analysis revealed that these DEGs were primarily enriched in viral infection and immune differentiation pathways, including “Human T-cell leukemia virus 1 infection”, “Epstein–Barr virus infection”, “Human immunodeficiency virus 1 infection”, “Th1/Th2 cell differentiation”, “and Th17 cell differentiation” (**Figure 5C**). GO enrichment analysis further indicated that these DEGs were significantly involved in biological processes such as positive regulation of cytokine production and leukocyte cell−cell adhesion (**Figure 5D**). Consistently, GSEA results demonstrated that most hallmark gene sets were significantly activated, including antigen processing and presentation, Th17 cell differentiation, and cell adhesion molecules (**Figure 5E**).

### 3.6 Impact of CDS risk score on immune infiltration in sepsis

**Figure 6:**
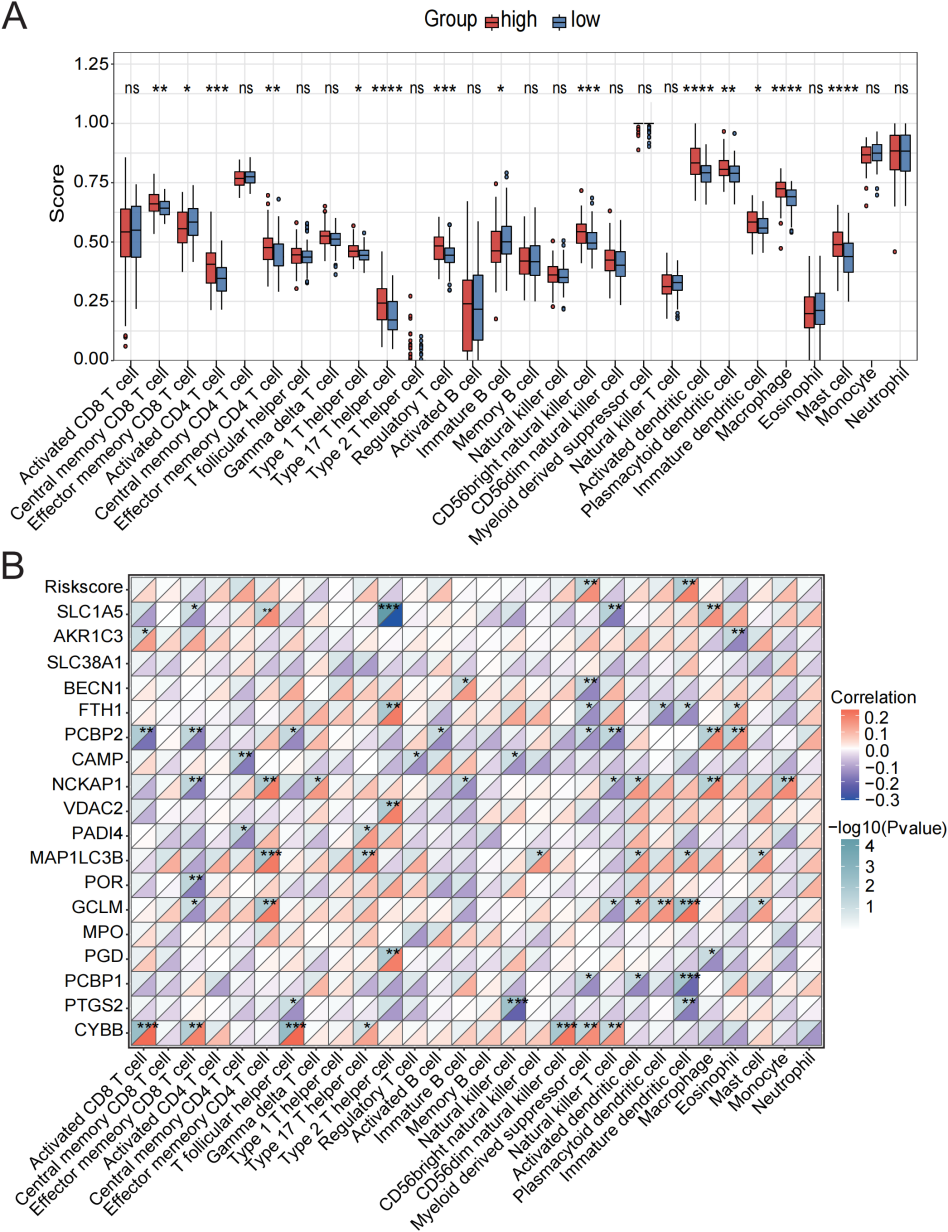
Assessment of immune cell infiltration in low-risk and high-risk groups. **(A)** Immune cell scores were assessed using ssGSEA analysis in the low-risk and high-risk groups. **(B)** Correlation analysis between the CDS risk score, core death genes, and various immune cell types. ns, not significant, *p < 0.05, **p < 0.01, ***p < 0.001, ****p < 0.0001.

To determine whether the CDS risk score accurately reflects the immune status in sepsis, we applied ssGSEA to evaluate immune cell infiltration. Distinct immune infiltration patterns were observed between high- and low-risk patient groups stratified by the CDS risk score. Among 22 immune cell types analyzed, 14 exhibited significant differences between the two groups. The majority of dysregulated immune cells were upregulated in the high-risk group, including central memory CD8^+^ T cells, T helper 17 cells, regulatory T cells, activated dendritic cells, macrophages, and neutrophils (**Figure 6A**, **Supplementary Table 10**), suggesting substantial alterations in the immune microenvironment among high-risk individuals. In contrast, effector memory CD8^+^T cells and immature B cells were significantly downregulated in the high-risk group.

Correlation analysis further revealed strong associations between the core cell death-related genes and various immune cell subsets (**Figure 6B**, **Supplementary Table 11**). Specifically, CYBB showed the strongest positive correlations with activated CD8^+^T cells, T follicular helper cells, and CD56 bright natural killer cells; GCLM and MAP1LC3B were positively associated with effector memory CD4^+^ T cells and immature dendritic cells; NCKAP1 exhibited strong positive correlations with NK cells, macrophages, and mast cells. In contrast, PCBP2 was negatively correlated with activated CD8^+^T cells, effector memory CD8^+^T cells, and natural killer T cells. Additionally, PTGS2 showed significant positive correlations with NK cells and immature dendritic cells. The CDS risk score itself was significantly positively correlated with myeloid-derived suppressor cells and immature dendritic cells.

### 3.7 Monocytes exhibit dominant activation of multiple PCD pathways in sepsis

**Figure 7:**
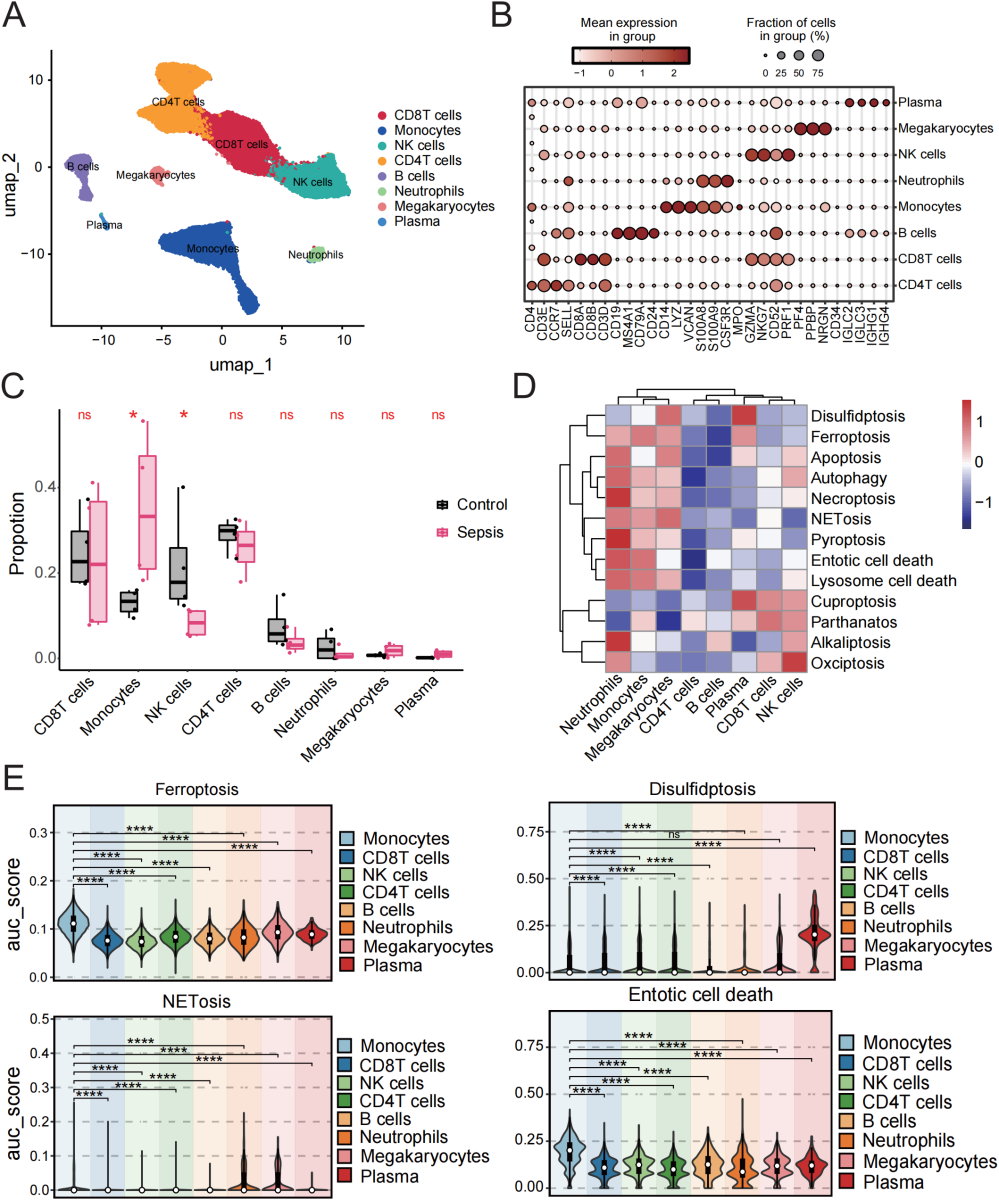
Monocytes exhibit dominant activation of multiple PCD pathways in sepsis. **(A)** 8 cell types were categorized using cell type-specific markers. **(B)** Dot plot showing the average expression levels of selected genes in each cluster. **(C)** Box plot comparing the proportions of immune cell subsets between sepsis patients and controls. **(D)** Heatmap of GSVA scores for 13 types of PCD in different cell types. **(E)** Comparison of AUCell scores of the cell death gene set between monocytes and other cell types. ns, not significant, *p < 0.05, ****p < 0.0001.

To further investigate the interplay between PCD and immune microenvironment alterations in sepsis, we analyzed a publicly available scRNA-seq dataset GSE217906 of PBMCs (https://www.ncbi.nlm.nih.gov/geo/query/acc.cgi?acc=GSE217906). Based on canonical marker genes, we manually annotated and identified the major immune cell types, including CD4^+^T cells, CD8^+^T cells, monocytes, natural killer (NK) cells, megakaryocytes, plasma cells, B cells, and neutrophils (**Figures 7A, B**).

Analysis of cell proportion revealed a markedly increased percentage of monocytes and a significantly reduced proportion of NK cells in septic patients compared to healthy controls (**Figure 7C**). We next examined the single-cell expression patterns of 18 core cell death-related genes identified from bulk transcriptomic analysis. Most of these genes exhibited significant differential expression between healthy individuals and sepsis patients and were predominantly expressed in the monocyte population (**Supplementary Figure 5**).

To analyze pathway-level activity at the cell cluster level, we first aggregated single-cell expression into pseudobulk matrices for each annotated immune cell type. Using these pseudobulk profiles, we performed GSVA to calculate enrichment scores for 13 PCD pathways. The results indicated a global activation of most PCD pathways (**Figure 7D**), with distinct activation patterns across different immune cell types. Specifically, entotic cell death, ferroptosis, NETosis, and lysosome-dependent cell death were more active in monocytes; neutrophils showed elevated activity in alkaliptosis, pyroptosis, necroptosis, apoptosis, and autophagy; while NK cells exhibited moderate activation across multiple pathways. In contrast, most PCD pathways were negatively enriched in CD4^+^T cells and B cells.

Subsequently, we calculated AUCell scores for four key cell death pathways—ferroptosis, NETosis, entotic cell death, and disulfidptosis—identified from transcriptomic analysis, to assess pathway activity at the single-cell level, and visualized their activity across immune cell types using violin plots. The results demonstrated that all four pathways were significantly more active in monocytes compared to other immune cell populations (**Figure 7E**).

### 3.8 Metabolism and cell death pathway profiling in monocytes of sepsis

**Figure 8:**
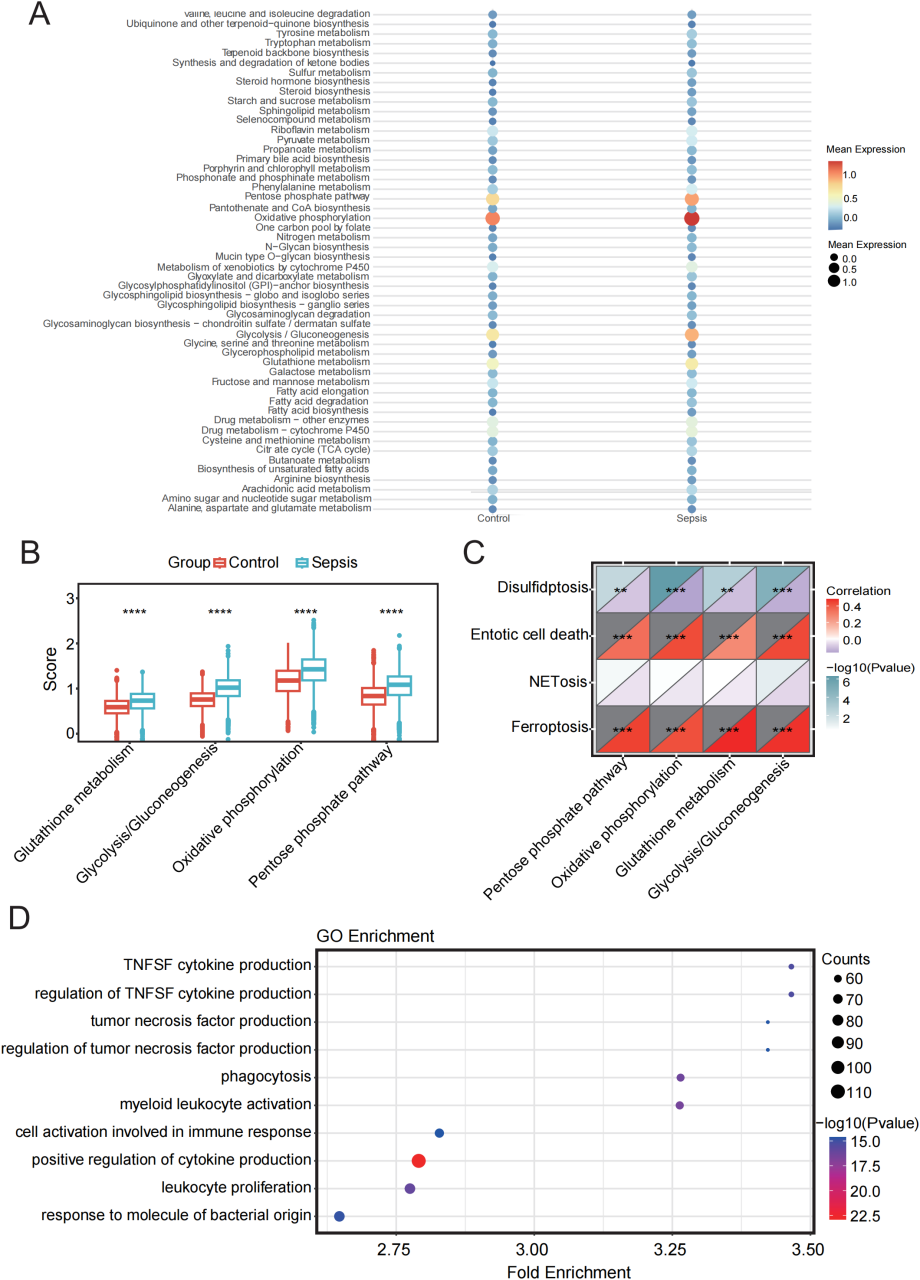
Metabolic pathway analysis and its relationship with PCD in sepsis and control groups. **(A)** Bubble plot showing enrichment of metabolic pathways in sepsis and control samples. **(B)** Box plot showing the comparison of four metabolic pathways between sepsis and control groups. **(C)** Correlation analysis between four metabolic pathways and four PCD pathways. **(D)** GO enrichment analysis of monocytes. **p < 0.01, ***p < 0.001, ****p < 0.0001.

Cell metabolism is fundamental to maintaining cellular function and survival. In disease states, cellular metabolism often undergoes reprogramming, which may be closely linked to cell death pathways. Therefore, we performed a metabolic analysis of monocytes. The results indicated that in four metabolic pathways: Glutathione-metabolism, Glycolysis/Gluconeogenesis, Oxidative-phosphorylation (OXPHOS), and Pentose phosphate pathway (PPP)—the sepsis group showed significantly higher scores than the healthy group, reflecting distinct metabolic features in sepsis compared to healthy conditions (**Figures 8A, B**).

Subsequently, we analyzed the correlation between the four cell death pathways and these four metabolic pathways. The results revealed a strong and highly significant positive correlation between ferroptosis and entotic cell death with the involved metabolic pathways, while disulfidptosis showed a significant negative correlation. NETosis exhibited minimal correlation with the metabolic pathways (**Figure 8C**).

GO analysis further revealed that the DEGs in monocytes were predominantly enriched in pathways related to the positive regulation of cytokine production, leukocyte proliferation, and immune cell activation, all of which are closely associated with immune responses. These findings underscore the pivotal role of monocytes in immune regulation and their functional interconnection with various immune cell types (**Figure 8D**).

### 3.9 Cell-cell interactions and signal pathways altered in sepsis

**Figure 9:**
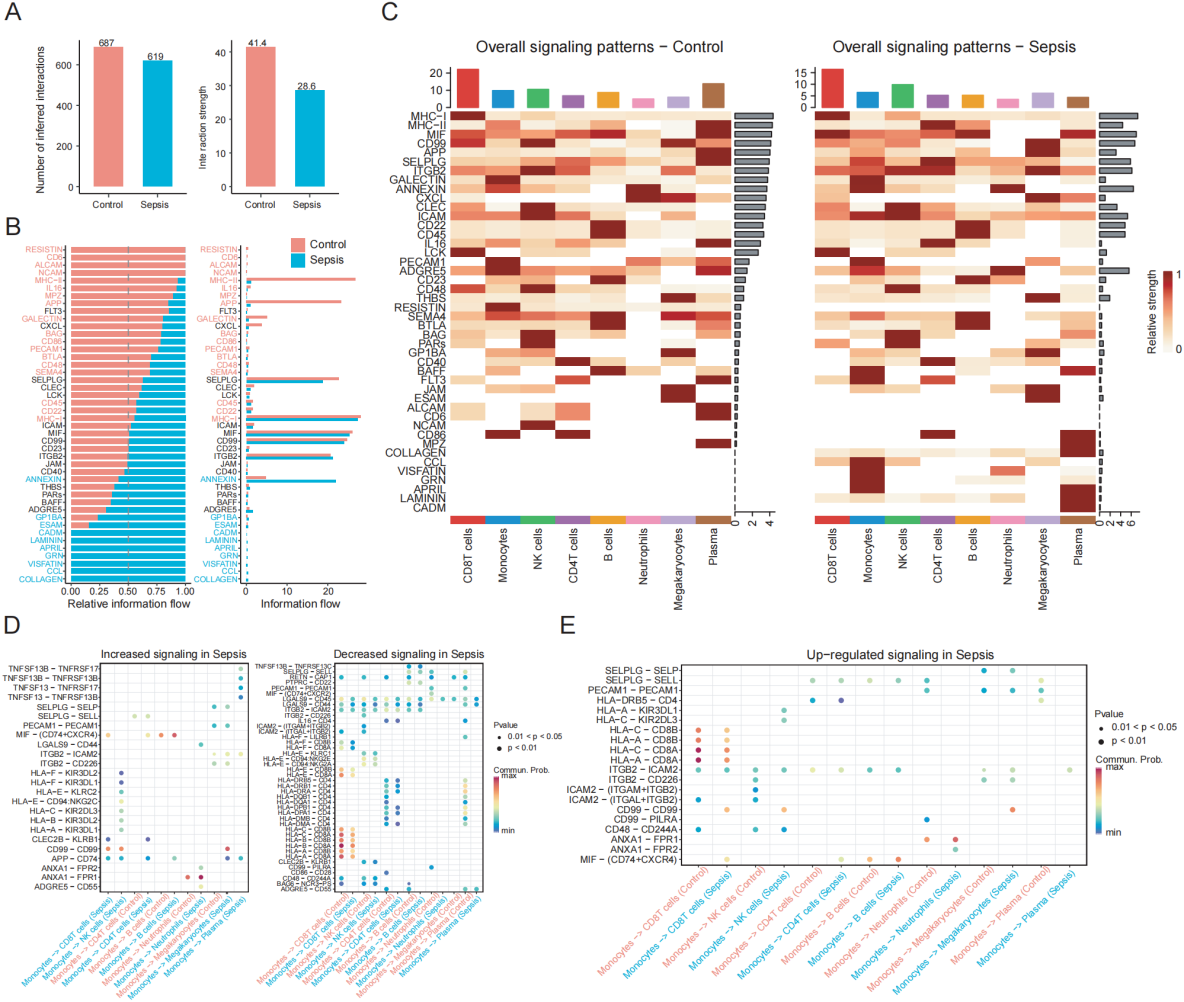
Altered monocyte-centric intercellular communication and signaling pathways in sepsis. **(A)** Bar plot showing the total number and overall strength of ligand–receptor interactions among PBMC subsets in the control and sepsis groups. **(B)** Cell communication analysis reveals differences in information flow across signaling pathways between the control and sepsis groups. **(C)** Heatmap shows the overall signaling patterns between the control and sepsis groups. **(D)** Dot plot shows ligand-receptor pairs with enhanced or weakened signaling in sepsis. **(E)** Dot plot shows upregulated ligand-receptor pairs between monocytes and other immune cell populations.

To determine whether monocytes regulate PCD pathways through interactions with other immune cell types, we performed CellChat analysis. Our results revealed a decrease in the total number and intensity of inferred interactions in sepsis patients, indicating impaired intercellular communication (**Figure 9A**). Changes in overall information flow revealed that multiple signaling pathways were significantly up- or downregulated under septic conditions (**Figure 9B**). Compared to the control group, signaling pathways such as ANNEXIN, CCL, and CADM showed enhanced relative information flow in sepsis samples, whereas classical immune-related pathways such as MHC-II, APP, IL16, and CD86 were markedly suppressed. Global signaling pattern analysis across different immune cell types further demonstrated that sepsis induced substantial alterations. Specifically, in monocytes, signaling pathways including ANNEXIN, CCL, FLT3, GRN, CADM, BAFF, APRIL, and VISFATIN were significantly upregulated in the sepsis group (**Figure 9C**).

At the ligand–receptor interaction level (**Figures 9D,E**), the MIF signaling axis exhibited enhanced outgoing signals from monocytes toward several immune subsets, including CD4^+^T and CD8^+^T cells, suggesting that this axis may serve as a central driver of inflammatory immune dysregulation. The HLA–KIR pathway, such as HLA-F–KIR3DL2 and HLA-F–KIR2DL3, was abnormally activated in NK cells, altering immune recognition signals transmitted from monocytes to NK cells and potentially increasing the risk of autoimmune damage and impaired immune surveillance. Similarly, the ANXA1–FPR1/2 axis and CD99–CD99 or CD99–PILRA signaling were specifically enhanced in sepsis, all of which are closely associated with cell migration, immunosuppression, and the regulation of adhesion molecules. Overall, monocytes in sepsis patients exhibited markedly altered interactions and signaling profiles, reflecting impaired intercellular communication and highlighting critical molecular changes involved in disease progression.

## 4 Discussion

This study systematically characterized the activation landscape of programmed cell death (PCD) pathways in sepsis and revealed their close association with immune–metabolic remodeling. We identified four key non-apoptotic pathways—ferroptosis, disulfidptosis, NETosis, and entotic cell death—as central processes in sepsis. Using 18 core genes derived from these pathways, we constructed a comprehensive CDS risk score that effectively discriminated sepsis patients from healthy controls, demonstrating robust diagnostic performance across both public datasets and an independent RNA-seq cohort. These findings highlight the diagnostic and biological relevance of cell death heterogeneity in sepsis.

Previous studies have confirmed that ferroptosis, disulfidptosis, and NETosis contribute to sepsis pathophysiology (38–41). In particular, our results are consistent with earlier reports showing that core genes involved in these pathways—such as FTH1 and GCLM, which are related to iron homeostasis and glutathione metabolism—were found to be upregulated in sepsis, promoting iron-dependent lipid peroxidation (42, 43). PADI4 and MPO contribute to the formation of NETosis: PADI4 facilitates chromatin decondensation by catalyzing histone arginine deimination, thereby promoting NET formation (44), while MPO, an enzyme produced by neutrophils and an essential component of the innate immune system, plays a role in microbial killing and has potential as a biomarker for distinguishing sepsis from non-infectious systemic inflammation (45). Together, these consistent findings validate the reliability of our transcriptomic-based identification of PCD activation in sepsis.

Of particular interest, entotic cell death, a non-cell-autonomous cell-in-cell death mechanism, has not been systematically investigated in the context of sepsis (46). This study is the first to reveal potential activation of the entotic pathway in sepsis patients and identifies two key genes associated with this process: BECN1 and CYBB. BECN1, a key autophagy initiator, regulates autophagosome formation and contributes to intracellular degradation and energy homeostasis. It also interacts with BCL-2 to modulate apoptotic pathways, and its dysfunction may disrupt cellular metabolism and energy balance, thereby influencing cell viability and sepsis progression (47, 48). CYBB (also known as NOX2), a central component of the NADPH oxidase complex, promotes ROS production and has been shown to be upregulated in sepsis, facilitating NETosis formation via oxidative stress (49). We further hypothesize that during entotic-like cell death, ROS accumulation may enhance the cytotoxicity of engulfing cells, with CYBB potentially mediating this killing effect. Future mechanistic studies—such as CYBB or BECN1 knockdown in monocytes—are warranted to clarify whether inhibition of these pathways alters metabolic remodeling or cytokine production, thereby establishing functional causality.

Ample evidence suggests that monocytes are among the most responsive immune cell subsets in sepsis, exhibiting increased peripheral abundance and functional reprogramming (50). Previous studies have mainly emphasized their immunosuppressive alterations under immune paralysis, such as downregulation of HLA-DR, impaired antigen presentation, and cytokine secretion dysregulation (51, 52). Consistent with these findings, our single-cell transcriptomic analysis also showed a significant expansion of monocytes in sepsis. Further pathway enrichment analysis revealed that monocytes exhibited significantly higher enrichment scores for both ferroptosis and entotic cell death pathways compared to other cell types, suggesting their potential as key effector cells in these two PCD processes. Additionally, the activity of NETosis was higher in monocytes than in most lymphoid cell populations, while the disulfidptosis pathway was relatively inactive, indicating heterogeneity in their involvement across different PCD pathways. To further investigate the regulatory mechanisms, we generated a metabolic profile of monocytes in sepsis and found significantly upregulated pathways, including glycolysis, OXPHOS, and the PPP. Previous studies have shown that in the early stage of sepsis, monocyte metabolism shifts from OXPHOS to glycolysis to promote pro-inflammatory responses; whereas in the immunosuppressive phase, OXPHOS dysfunction is closely associated with cellular functional exhaustion (53, 54). Our correlation analysis showed that Ferroptosis and Entotic cell death pathways were significantly positively correlated with the aforementioned metabolic pathways, suggesting that metabolic reprogramming may influence cell death fate via modulation of ROS homeostasis and cellular energy status. Ferroptosis depends on iron accumulation and lipid peroxidation; enhanced glutathione metabolism and PPP activity may influence ferroptotic thresholds through regulation of NADPH and GSH levels. Entotic cell death, as a cell-in-cell death mechanism, is influenced by cellular metabolism, viability, and ROS accumulation, with dysregulated glutathione metabolism potentially impairing ROS clearance and promoting entosis. These findings suggest that metabolic–death coupling represents a central mechanism linking immune dysfunction and cellular fate in sepsis, warranting further functional exploration.

Furthermore, cell–cell communication analysis revealed a dramatic remodeling of monocyte signaling networks in sepsis, with specific activation of multiple ligand–receptor axes. Notably, the MIF–(CD74+CXCR4), HLA–KIR, ANXA1–FPR2, and CD99–CD99 signaling pathways were significantly upregulated in sepsis. As observed in previous studies, the MIF axis was prominently enhanced in signaling from monocytes to CD4^+^/CD8^+^ T cells, suggesting its central role in driving inflammatory dysregulation (55, 56). Interactions such as HLA-C/KIR2DL3 and HLA-A/KIR3DL1 deliver inhibitory signals that suppress NK cell cytotoxicity, potentially facilitating immune evasion by pathogens (57). Although the ANXA1–FPR2 axis typically mediates anti-inflammatory responses under homeostasis, persistent stimulation may cause receptor desensitization, resulting in excessive neutrophil activation. We observed abnormal strengthening of this axis in monocyte–neutrophil interactions, implicating its role in failed inflammation resolution. CD99–CD99, an adhesion-mediated signaling pathway, was also specifically activated, potentially enhancing intercellular contact to amplify immunosuppressive signaling (58, 59). Moreover, monocyte-specific upregulation of pathways involving ANNEXIN, CCL, FLT3, GRN, and VISFATIN suggests their dual roles in immune regulation and PCD pathway activation via metabolism–death coupling mechanisms. Notably, FLT3 and GRN may regulate Ferroptosis and Entotic cell death through lipid peroxidation and the mTOR–lysosome axis, respectively. VISFATIN, a rate-limiting enzyme in NAD^+^ biosynthesis, may exert bidirectional effects in PARP1-dependent cell death.

Despite these promising findings, several limitations should be acknowledged. First, the sample size of the in-house RNA-seq cohort was relatively small, which may limit the generalizability of the CDS model. Second, the predictive performance of the risk score for 28-day mortality was modest, suggesting that additional clinical variables may be needed to improve prognostic accuracy. Future studies with larger, multicenter cohorts are warranted to validate the robustness and clinical utility of the proposed model.

## 5 Conclusions

This study systematically elucidates the activation landscape of 13 PCD pathways in sepsis by integrating bulk and single-cell transcriptomic analyses. Four non-apoptotic pathways—ferroptosis, disulfidptosis, NETosis, and entotic cell death—were significantly upregulated and strongly correlated with immune cell infiltration, suggesting their prominent roles in sepsis pathophysiology. Based on 18 core cell death–related genes, we constructed a CDS risk score model that demonstrated outstanding diagnostic performance in both publicly available microarray datasets and our independently generated high-throughput sequencing data. Further single-cell analyses revealed that monocytes exhibit dominant activation of multiple PCD pathways and serve as key effectors at the intersection of cell death, metabolism, and intercellular communication. In particular, metabolic reprogramming in monocytes was closely associated with ferroptosis and entotic cell death, highlighting the importance of metabolic-death coupling. Moreover, monocyte-specific ligand–receptor interactions, including the MIF–CXCR4, ANXA1–FPR2, and HLA–KIR axes, were significantly altered, indicating potential mechanisms for immune dysregulation and impaired resolution of inflammation. Collectively, these findings identify critical molecular features and cellular mechanisms underlying sepsis progression and offer a framework for future biomarker discovery and precision immunotherapy targeting PCD–metabolism–communication networks in sepsis.

## Data Availability

The sequencing data generated in this study were deposited at the National Center for Biotechnology Information’s Sequence Read Archive (SRA) under accession number PRJNA1172003.

## Supplementary material

Supplementary Figure 1 Batch correction. **(A, B)** PCA of datasets after **(B)** and before **(A)** batch correction. **(C, D)** Box plots of datasets after **(D)** and before **(C)** batch correction.

Supplementary Figure 2 Identification of core PCD genes. **(A)** Venn diagram analysis of intersection between DEGs and entotic cell death gene set. **(B)** Venn diagram analysis of intersection between DEGs and ferroptosis, **(C)** netotic cell death, or **(D)** disulfidptosis gene set. **(E)** Analysis of gene importance in random forest models.

Supplementary Figure 3 Calibration curve analysis (CCA) and decision curve analysis (DCA) of the CDS model across datasets. **(A, B)** Training cohort CCA and DCA. **(C, D)** External validation cohort (GSE69528) CCA and DCA.

Supplementary Figure 4 Predictive performance of the CDS risk score for clinical outcomes. **(A)** ROC analysis of the CDS risk score for distinguishing patients with high SOFA scores. **(B)** ROC analysis of the CDS risk score for predicting 28-day mortality in sepsis.

Supplementary Figure 5 Expression patterns of 18 core PCD related genes across major immune cell types in the control and sepsis groups at the single-cell level.
