# Supplementary figures and images for: Comprehensive analysis of the role of diverse programmed cell death patterns in sepsis
Source: Front Immunol. 2025 Nov 19;16:1685533. doi: 10.3389/fimmu.2025.1685533 (PMC12672461; doi:10.3389/fimmu.2025.1685533)

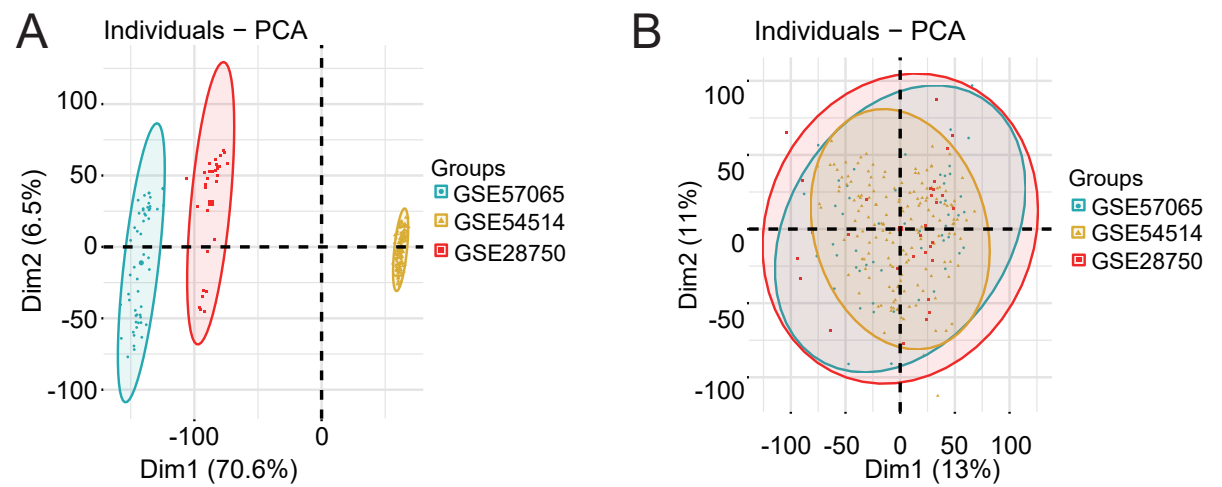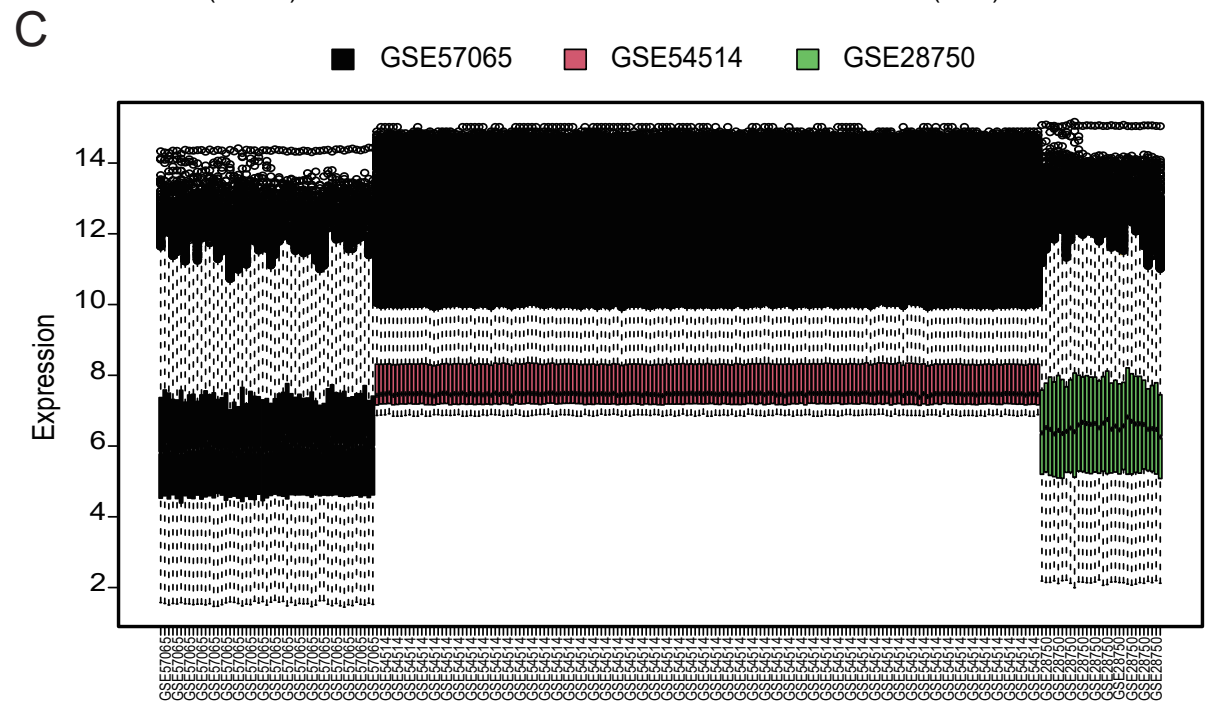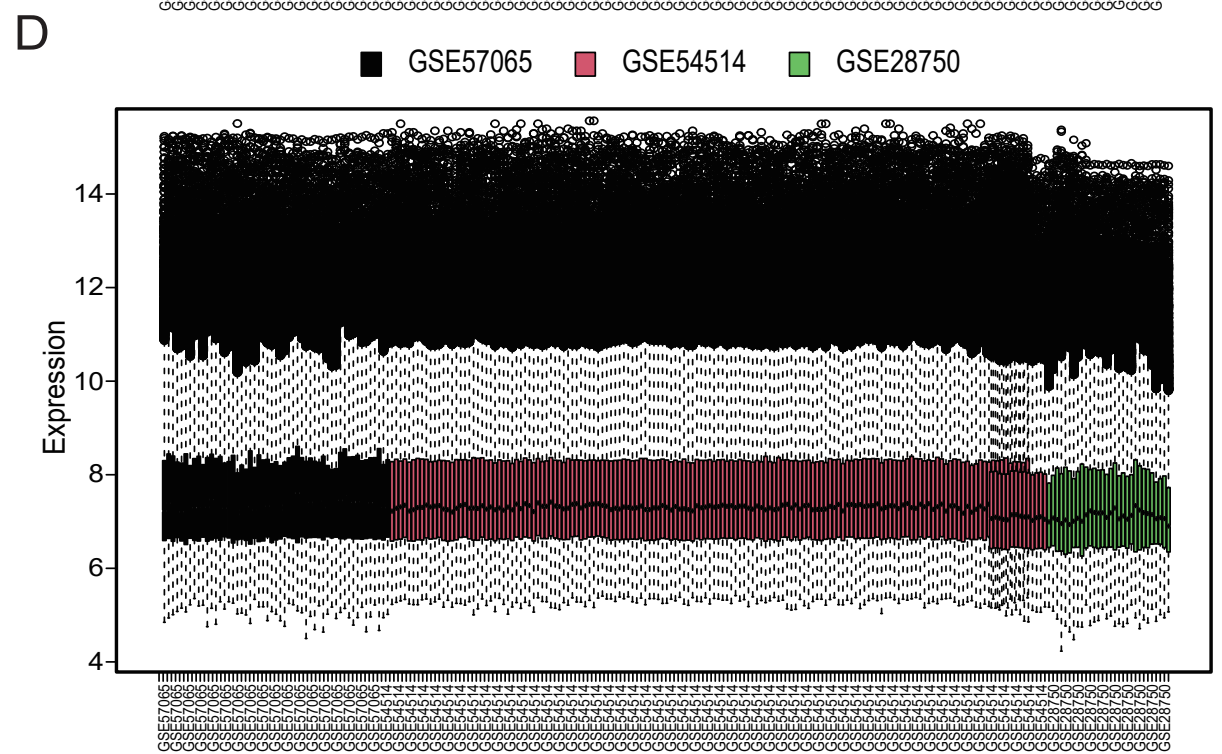

Supplement: Supplementary Figure 1 — Batch correction. (A, B) PCA of datasets after (B) and before (A) batch correction. (C, D) Box plots of datasets after (D) and before (C) batch correction. [file Image1.pdf]

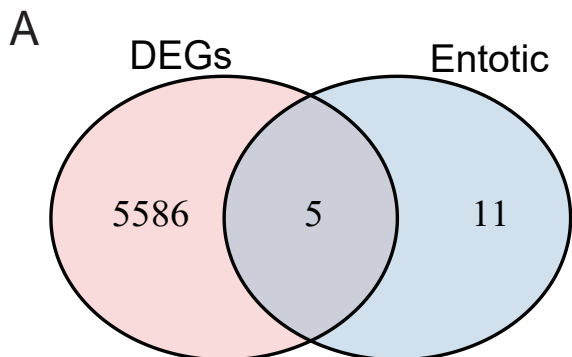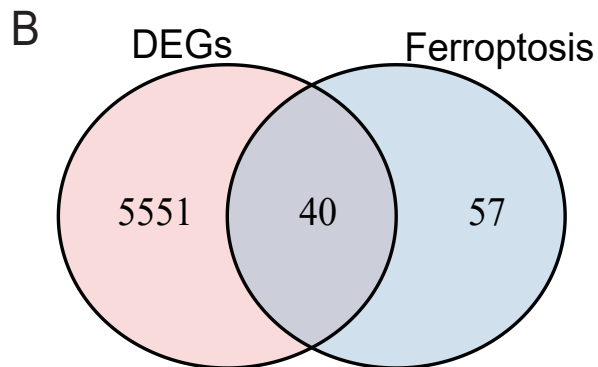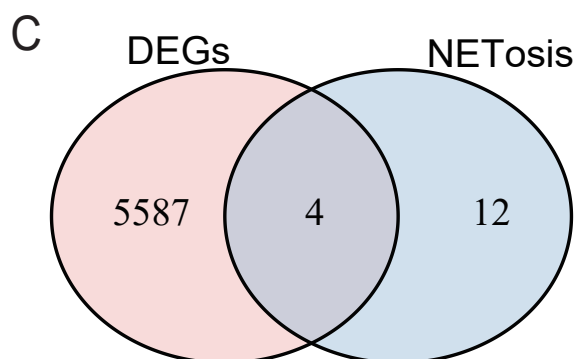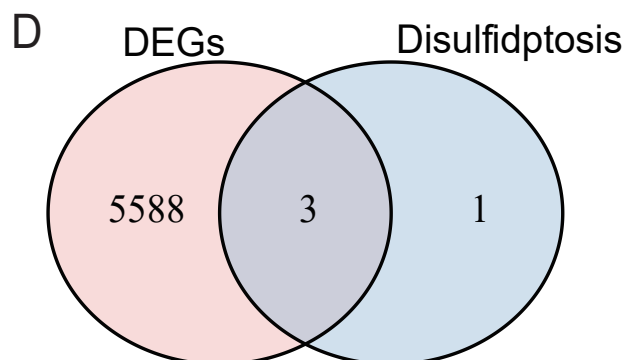

**E**

rf\_model

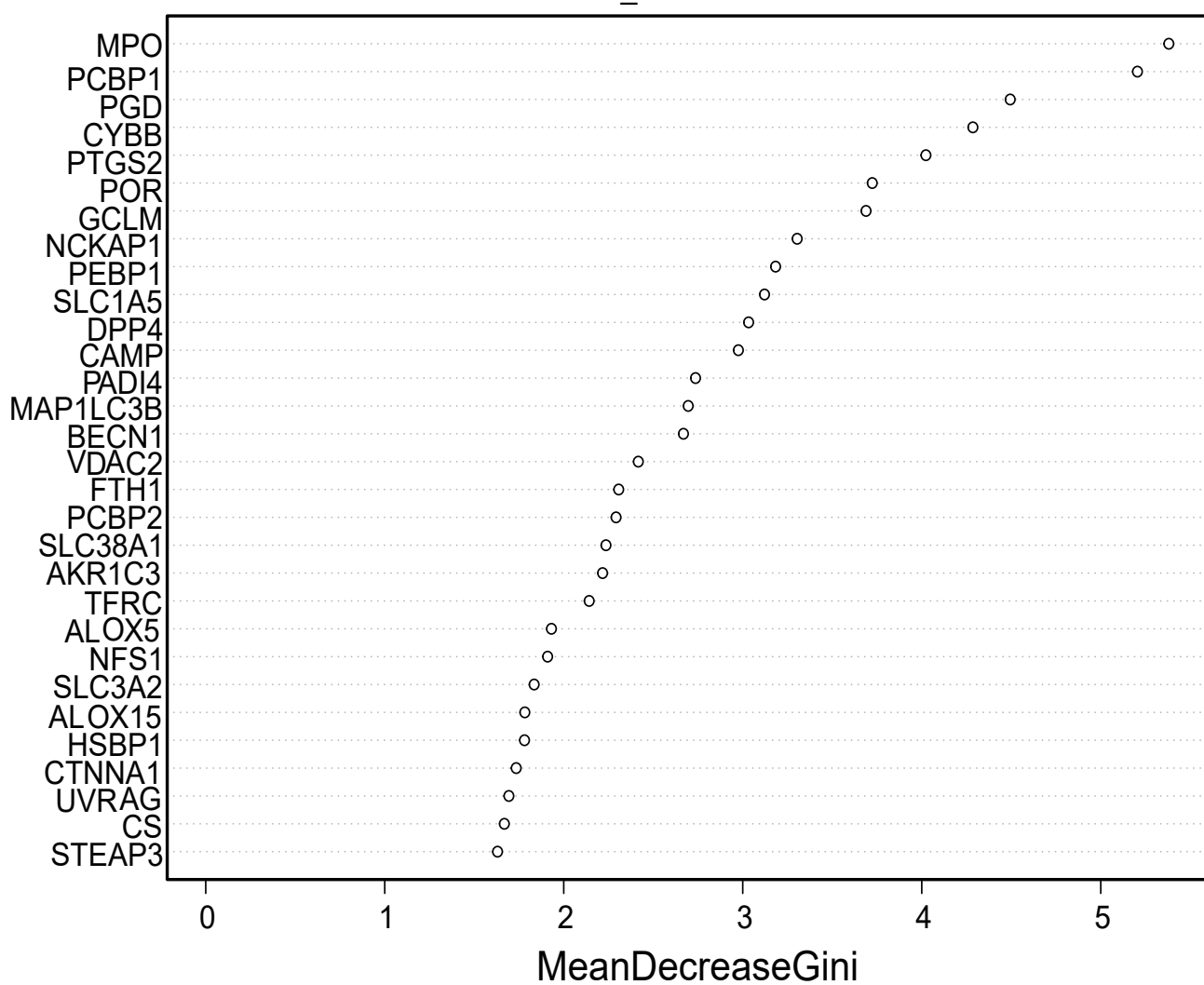

Supplement: Supplementary Figure 2 — Identification of core PCD genes. (A) Venn diagram analysis of intersection between DEGs and entotic cell death gene set. (B) Venn diagram analysis of intersection between DEGs and ferroptosis, (C) netotic cell death, or (D) disulfidptosis gene set. (E) Analysis of gene importance in random forest models. [file Image2.pdf]

A

## SOFA Validation

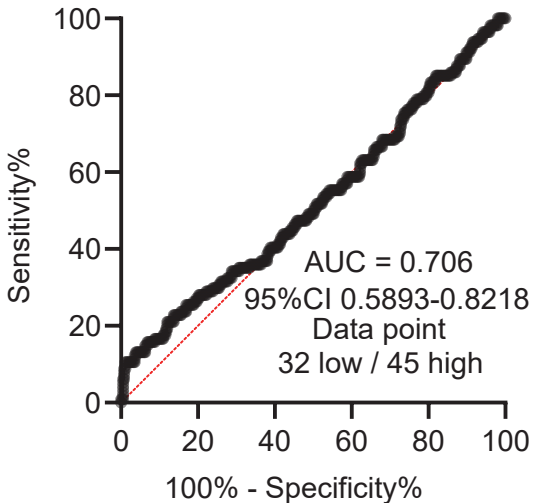

B

## 28 day mortality Validation

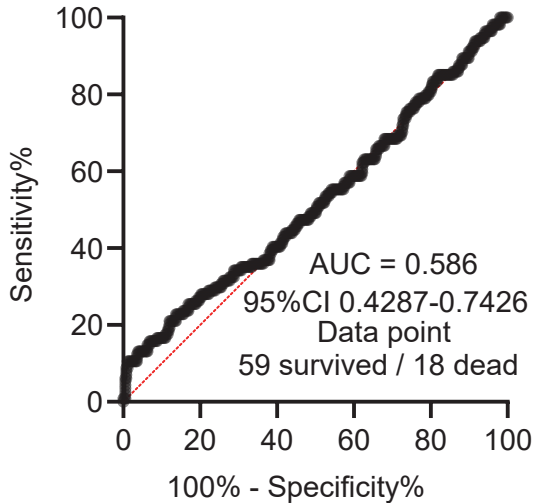

Supplement: Supplementary Figure 4 — Predictive performance of the CDS risk score for clinical outcomes. (A) ROC analysis of the CDS risk score for distinguishing patients with high SOFA scores. (B) ROC analysis of the CDS risk score for predicting 28-day mortality in sepsis. [file Image4.pdf]

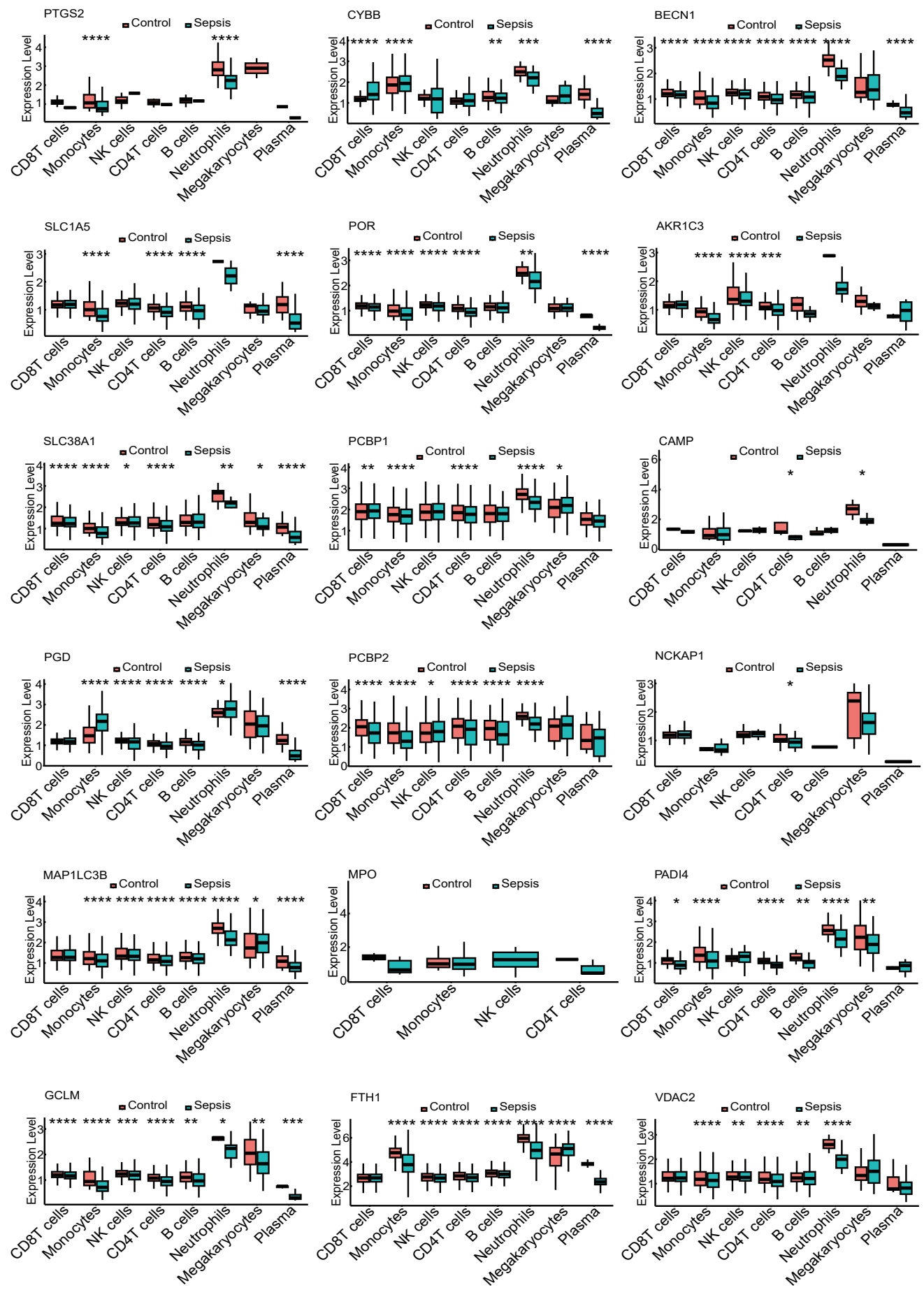

Supplement: Supplementary Figure 5 — Expression patterns of 18 core PCD related genes across major immune cell types in the control and sepsis groups at the single-cell level. [file Image5.pdf]
