# Supplementary material for: Comprehensive analysis of the role of diverse programmed cell death patterns in sepsis
Source: Front Immunol. 2025 Nov 19;16:1685533. doi: 10.3389/fimmu.2025.1685533 (PMC12672461; doi:10.3389/fimmu.2025.1685533)
Supplement: Supplementary Figure 3 — Calibration curve analysis (CCA) and decision curve analysis (DCA) of the CDS model across datasets. (A, B) Training cohort CCA and DCA. (C, D) External validation cohort (GSE69528) CCA and DCA. [file Image3.pdf]

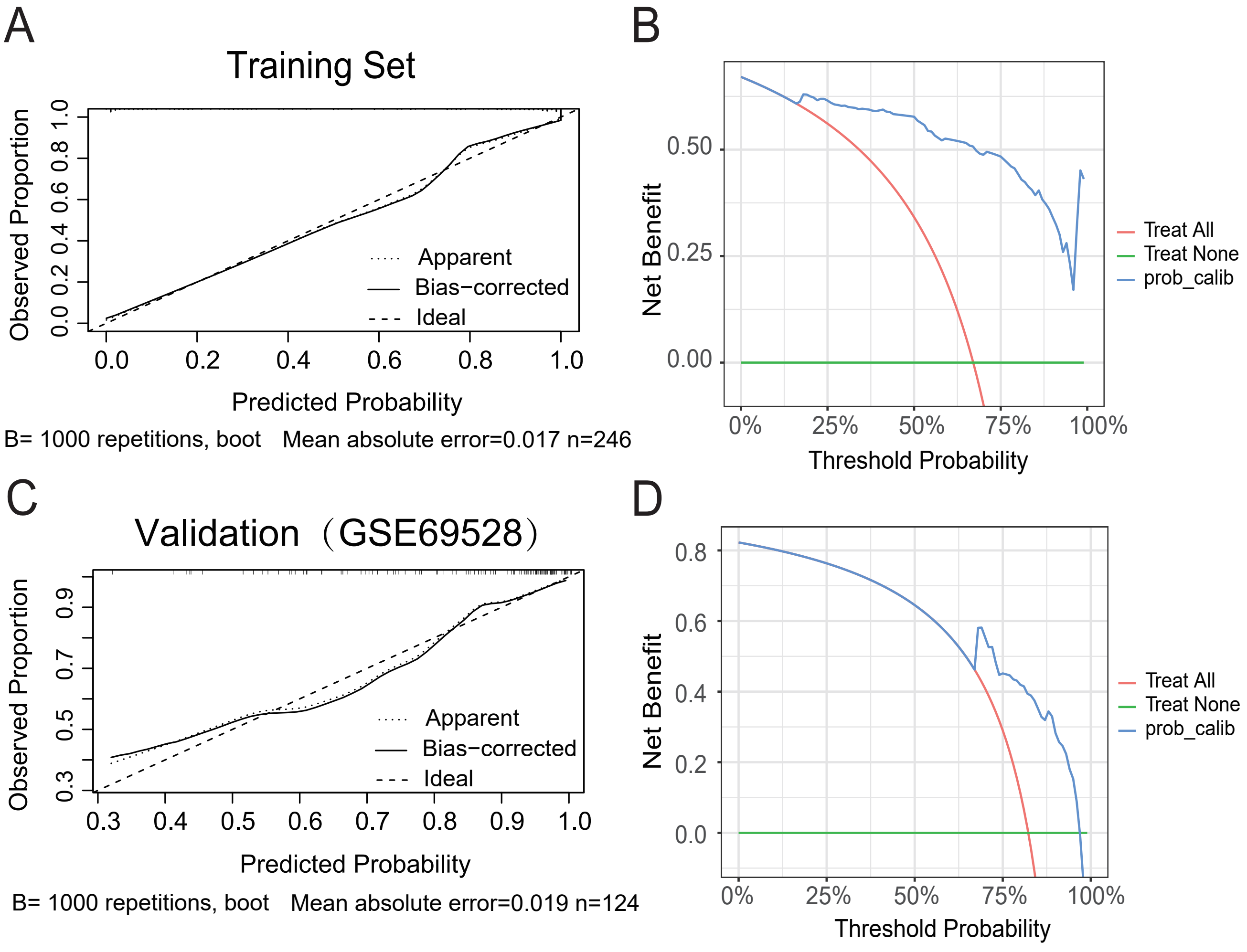
A
Training Set
Observed Proportion
0.0 0.2 0.4 0.6 0.8 1.0
Apparent
Bias−corrected
Ideal
0.0 0.2 0.4 0.6 0.8 1.0
Predicted Probability
B= 1000 repetitions, boot Mean absolute error=0.017 n=246
B
Net Benefit
0.50
0.25
0.00
0% 25% 50% 75% 100%
Threshold Probability
Treat All
Treat None
prob_calib
C
Validation（GSE69528）
Observed Proportion
0.3 0.5 0.7 0.9
Apparent
Bias−corrected
Ideal
0.3 0.4 0.5 0.6 0.7 0.8 0.9 1.0
Predicted Probability
B= 1000 repetitions, boot Mean absolute error=0.019 n=124
D
Net Benefit
0.8
0.6
0.4
0.2
0.0
0% 25% 50% 75% 100%
Threshold Probability
Treat All
Treat None
prob_calib
